# Pharyngolaryngeal semiology and prognostic factors in multiple system atrophy

**DOI:** 10.1007/s00405-022-07410-x

**Published:** 2022-05-05

**Authors:** N. El Fassi, Y. Gallois, S. Crestani, P. Fichaux-Bourrin, F. Ory, M. Fabbri, A. Pavy le Traon, V. Woisard

**Affiliations:** 1 Department of ENT in Toulouse, Hospital of Larrey, Toulouse, France; 2 Department of Neurology in Toulouse, Hospital of Purpan, Toulouse, France

**Keywords:** Multiple system atrophy, Dysphagia, Vocal fold paralysis, Abnormal movements of the larynx, Prognosis

## Abstract

**Introduction:**

Multiple system atrophy (MSA) is a rare degenerative neurological disorder in adults. It induces parkinsonian and/or cerebellar syndrome associated with dysautonomia. Pharyngolaryngeal symptoms are common. Our aim is to describe the Pharyngolaryngeal semiology on one hand, and to ascertain whether the presence of these symptoms represents a prognostic factor for MSA on the other.

**Methods:**

Thus, we carried out a retrospective, single-centre study, on a cohort receiving care at the centre of reference for MSA. The patients were referred for otorhinolaryngology assessment. The data was collected over the year 2020 with the help of computer software from the university hospital centre (UHC). Firstly, we described the Pharyngolaryngeal semiology specific to MSA by questioning patients, and by the results of nasofibroscopic examinations and swallowing tests. We then used multivariate analysis of variance to describe the prognostic factors of MSA progression (in UMSARS I and II points per month of progression) and survival (number of years between the first symptoms and death).

**Results:**

This study included a hundred and one patients and made it possible to define a Pharyngolaryngeal semiology profile of MSA, which is: a reduction in laryngeal mobility (primarily vocal cord abduction defects), abnormal movements (particularly at rest or when initiating a movement) and a defect in the protection mechanisms of the upper airways. The swallowing difficulties are moderate and the main mechanisms are delayed pharyngeal swallow and/or an oro-pharyngeal transport defect. In the multivariate analyses, the contributing factors are laryngeal anomalies, modification of solid food to fluid food and nutritional complication.

**Conclusion:**

ENT specialists should pay close attention to problems in the Pharyngolaryngeal dynamic and then consider a neurological cause. They can also itemize the clinical factors that could have a negative effect on the prognosis of the patient with MSA. Indeed, early detection makes it possible to provide care for respiratory and nutritional complications.

## Introduction

Multiple system atrophy (MSA) is a progressive neurodegenerative disease in adults belonging to the group of synucleinopathies which are characterized in MSA by abundant oligodendroglial intracytoplasmic inclusions. It is a rare pathology with an incidence of 0.6 to 0.7 cases for 100,000 inhabitants per year and with a prevalence that varies from 1.9 to 4.9 cases for 100,000 inhabitants [1]. It is a sporadic disease, although genetic forms have been described in some European and Japanese families [2]. It generally starts in the sixth decade of life.

Life expectancy varies between 6.2 and 10 years after the first symptoms [3–5]. The prognosis is poor and there is no curative treatment. The patients mainly die from sudden death, infections or mainly from aspiration pneumonia and urinary infections.

MSA affects the autonomic and central nervous systems: the patients affected present dysautonomia associated with parkinsonian and/or cerebellar syndrome and sometimes pyramidal symptoms. There are two phenotypes: parkinsonian (MSA-P) and cerebellar (MSA-C), depending on the predominance of the symptoms.

MSA induces also Pharyngolaryngeal disorders, which can appear early on and cause major disability. Although the Pharyngolaryngeal symptoms are present at all stages of the disease, this neurological pathology is poorly understood by ENT specialists and is difficult to detect during consultations.

The factors associated with a reduction in life expectancy are badly understood and vary between the different studies. However, there seems to be a link between the early occurrence of severe dysautonomia and the rapid progression of the disease [6]. Other independent factors that limit life expectancy have also been identified [7]: early autonomic failure (in the two years following the onset of the first symptoms), female gender, old age at diagnosis, a short interval between the diagnosis of the disease and the first symptoms attributed to the disease and the fact of not being admitted to a healthcare facility. Additionally, a prospective study carried out in 2020 by the French Centre of reference for MSA [8] (*n* = 261) found the prognostic factors of early death to be: the degree of incapacity in daily activities at the time of the examination, a short interval between the first symptoms attributed to the disease and the first medical visit in the reference center, progression of the severity of the orthostatic hypotension (measured by the UMSARS score: Unified Multiple System Atrophy Rating Scale). On the other hand, in this study, the following were not found predictive of excessive mortality: the MSA phenotype, the type of inaugural symptoms, and the age at which the diagnosis was made.

Although aspiration pneumonia (a likely consequence of swallowing difficulties) is a frequent cause of death in this disease, there are very few articles that describe the Pharyngolaryngeal prognostic factors specific to MSA. The literature deals above all with stridor, an additional diagnostic criterion for MSA that was first recognized in the 2008 consensus [9]. Although the impact of stridor on survival is uncertain, an early occurrence is known to be an independent marker of mortality and an occurrence when awake is evidence of an advance in the stage of the disease [10].

Additionally, a literature review of MSA produced in 2021 [11] relates the results of three studies that looked into the association between dysphagia and survival. It concluded that dysphagia (including swallowing aspiration/penetration events, coughing during and after eating or drinking with no specific characteristics described), is associated with poor survival. However, the results do not detail the severity nor the mechanism of the swallowing difficulty. Moreover, it was not determined whether the prognostic significance of dysphagia differs in function from the MSA phenotype.

In this work, we aim to describe the Pharyngolaryngeal symptoms of MSA (descriptive results) and the Pharyngolaryngeal prognostic factors impacting the progression of the disease and survival of MSA patients (analytical results).

## Materials and methods

### Study design and data collection

This is a descriptive, retrospective, single-centre study carried out at Toulouse University-Hospital Centre (UHC) and includes one hundred and one patients suffering from MSA. We used the cohort from the centre of reference in neurology service at Toulouse UHC, where the patient’s follow-up rate is annual. This is one of the largest cohorts of patients with MSA, with a prospective monitoring database shared by Toulouse UHC and Bordeaux UHC and registered at the CNIL (*Commission Nationale Informatique et Liberté*, n° 1 338 780; CCTIRS, n° 10.065).

To be included, the patients had to be adults with MSA confirmed by a neurologist and have had an ENT examination in the voice and swallowing unit (*unité de la voix et de la déglutition,* UVD) of UHC. After having extracted the patients corresponding to these criteria from the list, in compliance with the regulations, we sent a letter providing information about the study to the patients who were still alive.

Our study was carried out in the year 2020 using computer software to manage the Toulouse UHC patients’ files (ORBIS, Version 3.5 from 13/07/2016, AGFA HEALTHCARE France). This software manages UHC healthcare data via a healthcare data host. It enabled us to build a database comprising three sources of information: data extracted from the MSA centre of reference (BDAMS), data from the standardized ENT assessment reports produced by the specialists at UVD and archived video recordings of Pharyngolaryngeal examinations.

The data collected from the ***BDAMS*** included information on the population characteristics (gender, BMI, status and cause of death, medicinal treatments) and the disease characteristics (phenotype and type of MSA, age at onset of symptoms, age at diagnosis, age at death) and data on the severity of the disease based on the different clinical scales, particularly a scale defined specifically for MSA: UMSARS (Unified Multiple System Atrophy Rating Scale) [12]. These data are collected annually, if possible, in the context of follow-up care provision. UMSARS comprises 4 parts. It assesses the progression of the disease in terms of daily activities, motor and autonomic deficiency, and overall disability:

- UMSARS I (Historical Review), total score out of 48: assesses the functional consequences of the disease on daily activities and the severity of some non-motor symptoms;
- UMSARS II (Motor Examination), total score out of 56: based on the neurological examination and assessing motor disability;
- UMSARS III (Autonomic Examination): assesses orthostatic hypotension;
- UMSARS IV (Global disability scale): global disability scale. It varies from 1 (completely independent) to 5 (totally dependent, bed-ridden).

We only retained parts I (daily activities) and II (motor examination), which are generally used to monitor the severity of the disease [8]. To obtain the progression kinetics, we looked for and selected two scores in the BDAMS:

- UMSARS^ent^: established during the consultation with a neurologist that was the closest in time to the ENT consultation. The maximum interval between the neurological and ENT consultations to define UMSARS^ent^ was set at 100 days to limit the risk of an excessive clinical degradation in the interval between the two data collections.
- UMSARS^pre^: compiled during the preceding visit to the centre of reference in neurology. There was a maximum interval of 3 years between the two UMSARS collections.

The ***Pharyngolaryngeal data were*** compiled by analyzing the correspondence of ENT specialists. All patients were assessed in the same way, using a standardized assessment that specifically targeted swallowing. The report follows the same format and systematically includes patient complaints (dysphagia, dysarthria, dysphonia), medical history, feeding and nutritional status (malnutrition, change in diet, feeding support), lung and respiratory status (pneumopathies, respiratory distress), fiberendoscopic Pharyngolaryngeal assessment with a swallowing test and, if necessary, fluoroscopic evaluation of swallowing. The main complaints, feeding status and swallowing problems are presented on a checklist. Fiberendoscopic Pharyngolaryngeal assessment included the description and mobility of the pharyngo-larynx, the type of anomaly, the characteristics of the cough and swallowing difficulties with their mechanisms and severity (mild: few anomalies; moderate: “wrong way” swallowing and/or non-consequential stasis; severe: “wrong way” swallowing and/or stasis risking pulmonary and nutritional complications). This information was completed by scores from questionnaires addressing problems with the voice, phonation and deglutition, respectively, the Voice Handicap Index (VHI: 30 items ranging from 0 for “Never” to 4 for “Always” or a total score of 120) [13] validated in French [14] and based on the same principle: the Phonation Handicap Index (PHI, 15 items and a total out of 60) [14, 15] and the Deglutition Handicap Index (DHI, 30 Items and a total out of 120) [16, 17].

We also collated the treatment of ventilation complications (non-invasive ventilation/NIV, tracheotomy, continuous positive airway pressure/CPAP).

To finish, because we usually recorded the nasofibroscopic examination, we searched through the UVD’s digital archives the video recording for details of the examinations of the patients included. Out of the 101 patients, we only found 22 video recordings. These examinations were reread by two ENT specialists, who had no knowledge of the patients’ medical data using a report form describing the morphological and dynamic aspects of the pharyngo-larynx, and the characteristics of the swallowing difficulties.

### Statistical analysis

The descriptive results of the quantitative data are presented in the form of means and standard deviations (± SD) for the data following a normal distribution, and in the form of medians with their limits (minimum, maximum) or their interquartile ranges (Q1, Q3) for the data not following a normal distribution. For the qualitative data, the results are expressed in the form of percentages and frequencies of modalities with their confidence intervals [CI95%].

The descriptive data analysis was carried out for all the subjects since they all underwent an ENT consultation at Toulouse UHC, i.e. 101 patients with variations linked to the missing values: all the parameters were not systematically recorded despite the standardized data collection sheet. Missing data are less frequent for the Pharyngolaryngeal complaints (missing data for six patients, *n* = 95) and nasofibroscopy descriptions (missing data for seven patients, *n* = 94). They are more important for the questionnaires (respectively, for VHI, PHI, DHI: *n* = 68, 57, 74).

The distribution of the quantitative variables was analyzed by applying the Shapiro–Wilk test, and the variance using the *F* test. The normally distributed values of homogenous variance were compared using the unpaired parametric *t*-test, and the normally distributed values of non-homogeneous variance were compared using Welch’s *t* test. The not normally distributed values were compared using the Mann–Whitney *U* test. The analysis of the correlation between the quantitative variables was carried out by calculating Pearson’s r for the normally distributed variables, otherwise Spearman’s rank correlation was used. The qualitative variables were compared with the Chi-square test and Fisher’s exact test for small numbers.

The analytical description of the results crossing information from the BDAMS and data from consultations was done for the population whose interval between ENT consultation and disease information collection was less than or equal to 100 days, i.e. 75 patients. The integration of the qualitative and quantitative explanatory variables, to explain the chosen prognostic criteria for the disease, was carried out by using the multivariate analysis of variance (ANCOVA), taking into account the duration of the disease. The level of significance was set at 95% (*α* = 0.05).

After a collegial discussion between the neurologists and the ENT, and in view of the previous publications of the MSA Reference Centre [8, 18], these prognostic criteria were:

- the speed of disease progression: determined by the difference in the UMSARS I + II scores between the ENT consultation, UMSARS^ent^, and the preceding neurological consultation, UMSARS^pre^, accounting for the interval between the two measurements. We were able to perform the bivariate analysis on 75 patients and the multivariate analysis on 33 patients because of the accumulation of missing data linked to the number of variables introduced in the model.
- survival: determined by the number of months of progression between the date the symptoms appeared and the date of death. The multivariate analysis was done on 50 patients, which corresponds to the number of patients who had died.

The data were analyzed with XLStat version 2021.2.2 software.

## Results

### Selection of the population

Using the MSA centre of reference’s database (*n* = 301), we selected one hundred and one patients who had undergone an ENT examination at the UVD. Among this population, 75 patients had an interval of under 100 days between the ENT speech pathologist assessment and the closest neurological assessment.

### Descriptive results

#### I. General information

**Fig. 1:**
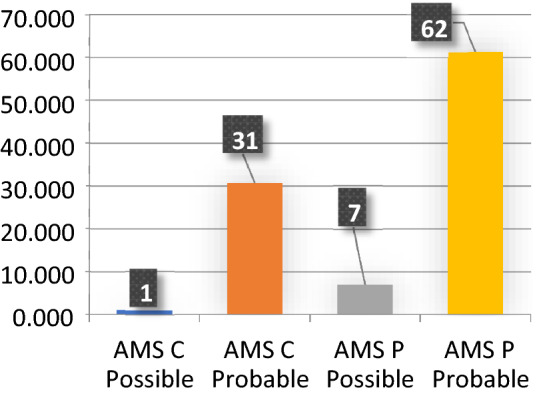
Phenotype and Diagnosis of MSA (number)

Fifty-five women and 46 men with a mean age of 65.5 years at diagnosis were included. The patients were identified as probably or possibly having MSA by the neurologists according to the diagnostic criteria established in 1998 by Gilman and colleagues and revised in 2008 [9]. Nearly 70% of the cases were parkinsonian forms (Fig. 1). Age at onset of symptoms was 59.5 ± 7.5 years. Age at diagnosis was 63,5 ± 8. Sixty-eight patients were dead during the follow-up of the cohort at a mean age of 68.2 ± 7.7.

The causes of death were not clearly identified for the majority of the patients. The main causes found were: sudden death (8.2%), aspiration pneumonia (16.5%) and overall alteration of the general condition (10.2%).

**Fig. 2:**
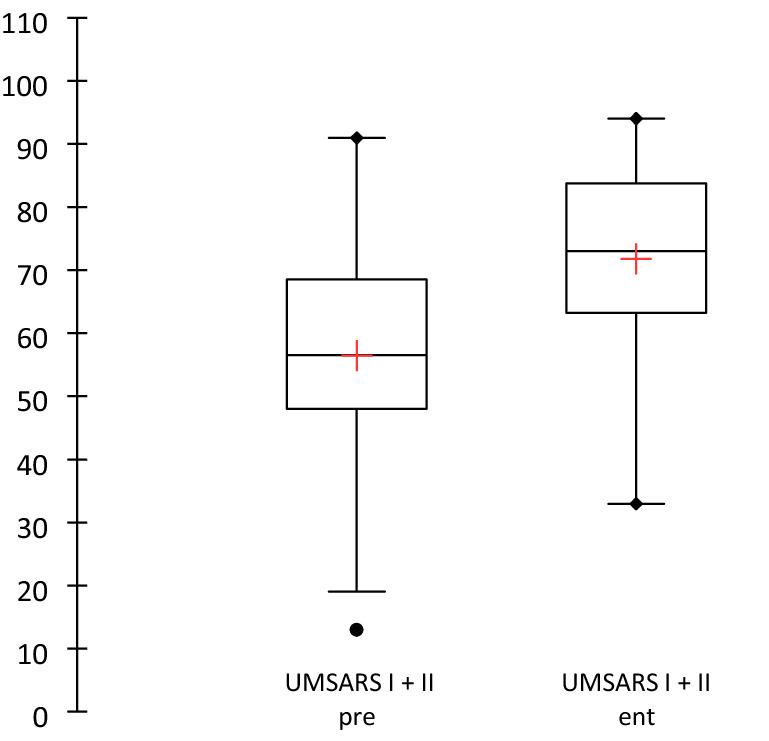
Progression of the UMSARS I + II score (The horizontal black line in the boxplot represents the median, and the red cross the mean)

The mean for UMSARS^ent^ was 71.83 ± 14.5. For 67 subjects, UMSARS^pre^ was collected: 56.71 ± 16.7. On average, 15 months ± 7.1 (Q1: 10, Q3: 19.5) passed between the two UMSARS data collections. The aggravation was statistically significant (*p* < 0.0001) (Fig. 2).

#### II. Pharyngolaryngeal complaints

The patients’ complaints are listed in Table 1.

**Table 1:** Pharyngolaryngeal complaints

Data *n* = 95	Percentage	CI 95%
Speech problems (voice and/or articulation)	17.8	10.4–25.3
Dysarthria	45	34.2–54.2
Dysphonia	21	12–28
No complaints	34	26.2–45.4
Difficulty swallowing^a^	32.7	23.5–41.8
Oral-phase swallowing	30.9	21.51–40.19
For liquids	60.6	50.76–70.52
For solids	47.9	37.77–57.97
Speech problems and difficulty swallowing^a^	43.6	33.9–53.2

^a^Some patients had oral-phase swallowing problems, for liquids and solids, all at the same time, which is why the totals for swallowing difficulties do not equal 100%

The MSA phenotype was not associated with the type of swallowing or speech complaint.

Swallowing difficulties for liquids tended to be more frequent than for solids (*p* = 0.09). The phenotype did not appear to influence the severity, whereas the DHI scores tended to be higher in MSA-P (mean of 35.6 ± 21 in MSA-C, and 46.2 ± 23.5 in MSA-P (*p* = 0.067)) with a higher frequency of oral phase swallowing problems in MSA-P (*p* = 0.063). 16.8% experienced “wrong way” swallows, 8% had blockages and there was one case of asphyxia, with no difference between the phenotypes.

In terms of speech production problems, the severity was ascertained by the questionnaire scores that surpassed the median, which for VHI gave a result of 67.5 (mean at 64.9 ± 24) out of 120 and for PHI a median at 40 (mean at 38.7 ± 14.6) out of 60.

There is a significant correlation (*p* < 0.0001) between speech production and swallowing difficulties gathered by the self-questionnaires. This correlation is higher with the VHI (*r* = 0.641) than with PHI (*r* = 0.586).

#### III. Description of the nasofibroscopy examinations

The table below (Table 2) summarizes the description of the nasofibroscopic examinations, as detailed in the ENT reports.

**Table 2:** Nasofibroscopic description

Data (*n* = 84)	Percentage	CI 95%
Observation of the Pharyngolaryngeal dynamic		
Isolated reduction in mobility	32	21.7–42.4
Isolated abnormal movements^a^	19.2	10.5–28
Reduction in mobility with spasticity (dystonia)	15.4	7.4–23.4
Reduction in mobility and abnormal movements	16.7	8.4–24.93
No anomalies	16.7	8.4–25
Observation of the swallowing test by fiberendoscopic or videofluoroscopic evaluation		
Delayed pharyngeal swallow	73.2	64.4–82
Oro-pharyngeal transport defect	53.6	43.7–63.5
Oral retention and/or initiation defect	41.2	31.4–51
Loss of protection mechanism	34.4	24.9–43.9
Oesophageal transport defect (visualised on the radioscopy)	34	24.6–43.4
Laryngeal closure defect	7.3	2–12.5
Rooling^b^	7.2	2.12–4
Assessment of the Cough (*n* = 64)^c^		
Effective	11	3.3–18.58
Impaired (little or no effectiveness, delayed cough)	18.7	9.2–28.31
None	70.3	59.11–81.5

^a^Tremors, abnormal movement of the arytenoids, paradoxical adduction movement on inspiration

^b^This was a form of oral-phase swallow initiation defect equivalent to gait festination described as specific to parkinsonian syndromes [8]

^c^Searched for by nasofibroscopy contact in the larynx or by observing the occurrence of laryngeal penetration

The MSA-P patients tended to present more abnormal movements, while the MSA-C patients presented more reductions in the mobility of the larynx (*p* = 0.143). Out of the 101 patients, only one had proven stridor at the time of examination.

All the patients have a swallowing disorder at the swallowing test. The degree of severity is moderate in the majority (53%), 20% had mild difficulty and 27% severe. 89% of patients presented an altered cough (impaired or no cough as described in Table 2) corresponding to a defect in the protection mechanisms of the lower airways. Laryngeal penetrations or aspiration tended to be more frequent in MSA-C (*p* = 0.079).

To be noted: less than half of the patients performed a videofluoroscopy of swallowing. 34% of these patients presented an esophageal transit anomaly.

#### IV. Nutritional and ventilation complications

These anomalies led to a change in diet (information missing for five patients). Indeed, 39.6% [CI 29.8–49.37] were forced to change their dietary practices for solid foods (blended, soft, fluid food), 11.5% presented a contraindication to oral feeding or maintained only “pleasure” feeding. Liquids on the other hand were contraindicated in 13% of cases and thickened in 13% of cases.

Nutritional support was sometimes required, with 7% of patients on dietary supplements, 22% on enteral feeding (nasogastric tube or gastrostomy) and 1% on parenteral nutrition. About enteral feeding, 72 patients had no indications for its introduction, 28 had an indication for a gastrostomy but 6 of these refused enteral feeding. On the other hand, we should stress that 25% of cases had a BMI < 21, and the mean BMI was 23.2 ± 4.15.

The complications of swallowing difficulties can be serious and fatal. By reading the ENT reports, we noted that 32% of the patients had pneumopathies. Their occurrence was not linked to age, BMI, ENT scores, the severity of the disease (reflected in the UMSARS scores), swallowing mechanism’s impairment nor aspirations. However, although it was not significant, we noted that among patients with pneumopathies, 17% had pneumopathies when their swallowing difficulties were mild, compared to 37% in cases of moderate and 37% in cases of severe difficulties. Additionally, of the patients lacking a protection mechanism, 40% had aspiration pneumonia, compared to 31% if this mechanism was effective. We noted, however, that dietary changes (for solids and liquids) were statistically linked to the preexistence of pneumopathy. Additionally, the introduction of CPAP was more frequent in patients who presented a broncho-pulmonary infection (*p* = 0.002).

In this cohort, no emergencies tracheotomies were performed but 18 patients required NIV, with a statistical link between the introduction of NIV and type C MSA (*p* = 0.016). Concerning sleeping troubles, 15 patients were given CPAP therapy.

#### V. Rereading the nasofibroscopic examination

Finally, we present below a Table 3 describing Pharyngolaryngeal troubles following a rereading of 22 videos by two ENT specialists.

**Table 3:** Results of the nasofibroscopic examination rereading (*n* = 22)

Variable	Number of missing values	Frequency by modality (%)	95% CI
Observation of the larynx			
Endo-laryngeal secretions	0	22.7	5.2–40.2
VC Atrophy	0	68.2	12.4–51.3
VC Mobility defect	0	90.9	78.9–100
Adduction paresis	0	27.3	8.67–45.9
Abduction paresis	0	81.8	65.7–97.9
Paradoxical adduction while sniffing	4	33.3	11.56–55.11
Phonation adhesion defect	2	45	23.2–66.8
Resting tremors	1	52.4	31–73.7
Intention/action tremors	2	70	49.9–90
Arytenoid flapping	2	40	18.5–61.47
Swallowing assessment			
Localised stasis	3	31.6	10.7–52.48
Extensive stasis	1	47.62	26.25–68.9
Diffused stasis	2	70	50.2–85
Mild stasis	3	52	25.6–64
Laryngeal penetration with immediate protection	1	33.3	13.17–53.5
Penetration before swallowing	1	42.85	21.7–64
Aspiration with immediate protection	1	14.3	0–29.3
Aspiration before pharyngeal trigger	1	23.8	5.6–42
Oral retention and/or initiation defect	3	36.8	15.5–58.5
Oral control defect	2	65	44–85.9
Oropharyngeal transport defect	2	90	76.85–100
Delayed pharyngeal swallow	1	90.5	77.9–100
Protection mechanism defect	6	68.75	46–91.5

In our study, we lack information about the voice. Our standardized assessment is more oriented toward swallowing disorders than for voice or speech disorders.

In particular, we noted that 91% of the patients had vocal cord mobility defects with a majority of abduction defects and that more than 50% had resting tremors (during respiration). Additionally, in pre-phonation, more than 50% of cases presented a concave aspect of one or both vocal cords.

At the swallowing test, the residues were diffused in 70% of cases and mild in 52% of cases. The main mechanisms of the swallowing difficulties were delayed pharyngeal swallow (DPS) and faulty oropharyngeal transport, which we have already observed.

The size of the airway was statistically linked with ventilation complications (*p* = 0.012 for NIV, *p* = 0.004 for CPAP), as well as with the severity of the swallowing (*p* = 0.014). Moreover, there was a weak but significant positive correlation between BMI and sthe ize of the airway (*r* = 0.38), or in other words, the lower the BMI, the smaller the size of the airway. Therefore, airway size appears to be globally related to the severity of the disease through its impact on respiration and nutrition.

### Analytical results

In the second phase, we analyzed the link between Pharyngolaryngeal troubles and disease progression for 75 patients (those with an interval between the ENT consultation and the UMSARS score assessment of ≤ 100 days) via two different criteria:

- the speed of disease progression: determined by the difference in the UMSARS I&II ^pre^ and ^ent^ scores, accounting for the interval between the two measurements (UMSARS points per month of progression);
- survival: determined by the number of months of progression between the date the symptoms appeared and the date of death.

For the speed of progression, the mean progression of the disease was 1.06 ± 0.62 points per month. The bivariate analysis was carried out for all of these 75 patients. The multivariate analysis included as explanatory variables all the parameters for which a difference appeared with a *p* < 0.2, which only allowed us to include 33 patients in the calculations. On this criteria, the parameters included in the multivariate analysis are upper aero-digestive tract complaints, laryngeal anomalies, severity of the swallowing difficulties, loss of protection mechanism, food modifications, and nutritional complications. The factors, which contribute to the speed of progression of the pathology, in the bivariate are laryngeal anomalies (*p* = 0.032) and modification of the texture of liquids from normal to thickened (*p* = 0.009). There is a trend but not significant contribution concerning the loss of protection mechanism (*p* = 0.062). In the multivariate analyses the contributing factors are laryngeal anomalies, modification of solid food to fluid food and nutritional complication with, respectively, *p* = 0.008, 0.013, 0.035 and a normalized coefficients at − 0.421 (CI 95% [− 0.724 − 0.119]), 0.482 (CI 95% [0.109 0.855]) and 0.336 (CI 95% [0.026 0.646]).

The survival was calculated for the 68 patients who had died. It was of 8.5 ± 2.7 years. The parameters with a *p* < 0.2, only allowed us to include 36 patients in the calculations. On this criterion, the parameters included in the multivariate analysis are only the time interval before diagnosis, laryngeal anomalies, severity of the swallowing difficulties and pulmonary complication as pneumopathies. In the bivariate analysis, only the time interval before diagnosis was statistically correlated with survival (*p* = 0.0001), movement anomalies of the vocal folds (*p* = 0.022) and pneumopathies (*p* = 0.042). In the multivariate analysis, the Pharyngolaryngeal factors that had an incidence on survival were: time interval before diagnosis (*p* < 0.0001, normalized coefficients = 0.515 [0.225 0.805]) and laryngeal movement anomalies (*p* = 0.022, normalized coefficients = − 0.421 [− 0.724 − 0.119]). The other factors were not associated with survival.

**Fig. 3:**
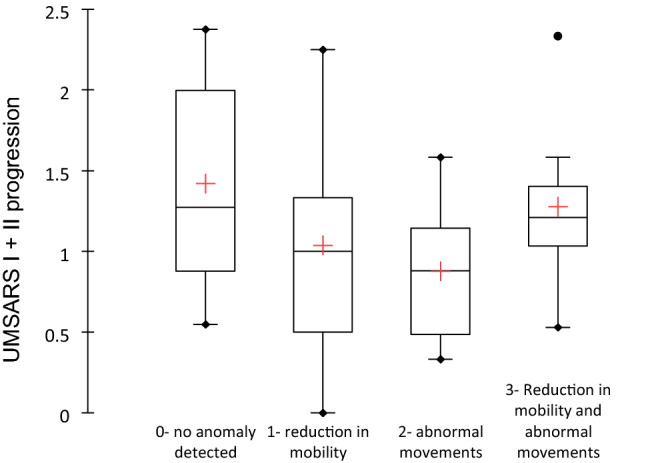
Variations in UMSARS I + II score in function to the laryngeal anomalies

Finally, the outcome regarding laryngeal anomalies seems paradoxical (negative correlations): when no anomaly was detected, the disease progressed quicker (Fig. 3) and the survival is shorter. This observation led to additional analyses highlighting two possible explanatory factors. The first is a significant difference between the time of diagnosis laryngeal abnormalities. The time of diagnosis being shorter when there is no abnormality detected and longer when a reduction in mobility and abnormal movements are observed (*p* = 0.032). The second is the absence of a significant difference between the severity of swallowing disorder or pneumopathies and the presence of laryngeal abnormality.

## Discussion

***As concerns epidemiology***, to our knowledge this is one of the retrospective studies with the greatest number of subjects to describe the situation of a population of MSA patients who underwent a standardized pharyngolaryngeal work-up. This is also the only one to include the notion of severity. The population in our study is representative of the European population in terms of the following parameters: gender, phenotype (predominance of MSA-P), treatments, age and interval before diagnosis [3, 8, 19].

However, the duration of the disease progression was shorter in our study: 8.5 years compared to 9.8 years for the NAAMS-SG, 9.3 for Foubert-Samier et al. [8]. The first reason for this difference is linked to the fact that the patients were recruited in a tertiary centre of reference, which improves the diagnostic precision but potentially increases the interval between the onset of symptoms and the first consultation. The second is linked to the main selection criteria of our population: having been referred by neurologists because of the presence of ENT symptoms.

***Concerning the laryngeal anomalies,*** the different studies [20] that pertinently describe the troubles found in MSA concur in identifying 4 groups of laryngeal anomalies:

- Uni or bilateral adduction paralysis while speaking
- Uni or bilateral abduction paralysis while sniffing
- Bilateral paralysis (shrinking of the laryngeal airway)
- Abnormal movement (arytenoid flapping, irregularities in frequency and amplitude, but with no adduction or abduction difficulty)

**Fig. 4:**
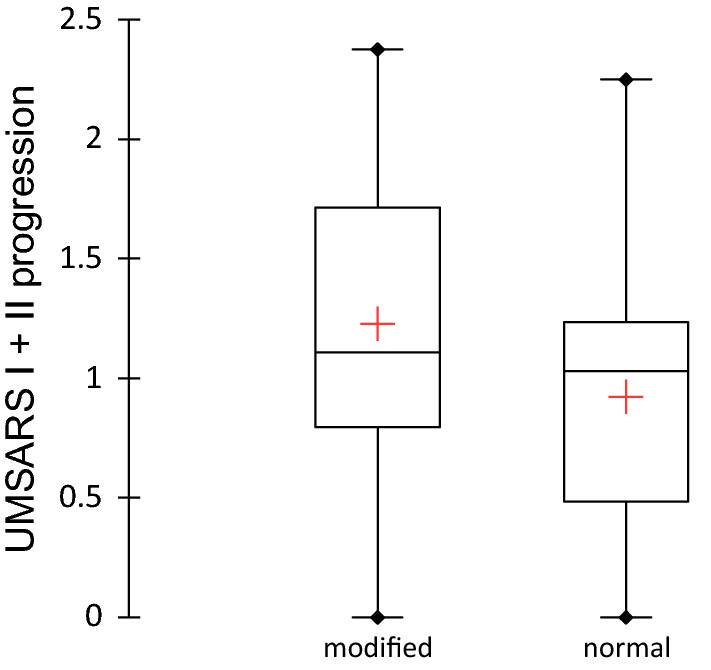
Solid food diet in function to disease progression

Grimaldi’s study [20] suggested that abnormal movements could be the first stage of MSA, followed by abduction problems and finally bilateral paralysis in the advanced stages. This analysis does not completely concur with the description of patients suffering from bilateral paralysis at different stages, present for more than one year before the diagnosis was made. Indeed, Tipton et al. [21] described four cases of C phenotypes where two required a tracheotomy before the diagnosis. But these cases were all of the C phenotype, and there was perhaps a delay in the diagnosis. This observation [20, 21] led the authors to emphasize the fact that a neurodegenerative disease of synucleinopathy type should be considered when confronted with vocal fold paralysis, and that an associated “rapid eye movement” (REM) sleep behaviour disorder or paradoxical disorder should be sought (Fig. 4).

The works comparing the vocal cord movement anomalies between MSA and Parkinson’s disease (PD) [18, 22] are particularly interesting to help differentiate the two pathologies during a nasofibroscopy. Although adduction paralyses are found in the two pathologies, the other troubles are almost exclusively observed in MSA. For Warnecke et al. [22], arytenoid fluttering or flapping and abduction defects are pathognomonic signs of MSA. This fact was corroborated by Gandor et al. [23], who explained that arytenoid fluttering could serve as a biomarker to differentiate between MSA and PD. These studies also confirm the importance of stridor, already identified as a factor for a poor prognosis in Krim and Yekhlef’s study (RR = 3.64; *p* = 0.006) [24].

The results of our study concur with the typology of laryngeal anomalies described. They confirm the prevalence of troubles “at rest” during respiration more particularly for inspiration (paradoxical adduction movement on inspiration or provocation by sniff test) and abduction paresis. We provide an additional piece of information to the literature: MSA-P patients tend to present more abnormal movements while MSA-C patients present greater reductions in larynx mobility.

However, one of the limitations lies in the lack of information on the timing of the onset of different symptoms and cannot determine the precocity and chronology of the Pharyngolaryngeal symptoms. Our study also produced results that appear contradictory.

The first concerns the low frequency of stridor collated in the ENT files. The reasons proposed are the standardization of the work-up (which does not include a specific section for stridor) and the fact that nighttime stridor is not systematically sought for and appears earlier than daytime stridor.

The second is the absence of tracheotomy despite the presence of bilateral laryngeal paralysis. We think that this is linked to an early systematic provision of non-invasive ventilation for all laryngeal diplegia in our service. Finally, the paradoxical result concerning laryngeal abnormalities may be related to the poor prognosis of early diagnosed forms, the absence of laryngeal abnormalities being associated with a shorter delay between the initial symptoms of the disease and the diagnosis. Another explanation may be the fact that UMSARS scores I and II tend to peak in severe forms. Indeed, we note that before consulting the ENT specialist, the UMSARS scores were generally already high (> 50 points) and the literature reports [25] that UMSARS scores progress rapidly at the beginning and then reach a plateau. This suggests that worsening of laryngeal abnormalities during disease progression will be difficult to describe in the absence of prospective follow-up including laryngeal assessment.

***As concerns swallowing difficulties,*** a consensus conference was organized in 2019–2020 [10] to reach a consensus on the diagnosis, prognosis and treatment of dysphagia in MSA (literature review of 27 studies). There, dysphagia was described as a frequent (31 to 78%) and invalidating symptom. Its appearance in the 5 years following the onset of motor symptoms is an additional diagnostic criterion. The swallowing difficulty mechanisms are often described in terms of stasis in UADT, laryngeal penetrations or “wrong way” swallowing, and rarely in more precise terms referring to physiopathological defects or mechanisms.

Overall, the most frequent observations were vallecular residues (68% and 89.8%) [24, 25], followed by penetrations/wrong way swallows (67.8%) [24] and residues on the posterior pharyngeal wall (66.1%) [24]. In our study, 76% of patients had residues in the vallecular or pyriform sinus and 56.1% experienced penetrations/inhalations, which is coherent with the literature.

The prevalence of the type of swallowing difficulty in function to the type of MSA was particularly studied at the consensus conference [10] and by Lee et al. [26]. They reported that in the early phase, the swallowing difficulties appeared more severe in MSA-P and mostly affected pharyngeal phase swallowing, whereas impaired oral-phase swallowing came earlier in MSA-C with more “wrong way” swallows. Additionally, the following oral phase swallowing symptoms were more frequent and more severe in MSA-P: swallowing difficulty, increase in length of meals, drooling, observation of lingual apraxia and vallecular residues. Our study confirms the prevalence of oral phase swallowing anomalies and their severity in MSA-P and notes the prevalence of impaired pharyngeal phase swallowing with a greater frequency of penetration/wrong way swallows in MSA-C. The fact that the DHI tends to be more severe in MSA-P (score mean of 35.6 ± 21 in MSA-C and 46.2 ± 23.5 in MSA-P) serves to confirm a greater severity for MSA-P. However, our work did not enable us to observe whether the oral phase swallowing troubles come earlier in MSA-C, and it would be interesting to confirm this information in the future.

Our cohort demonstrates the main mechanisms of swallowing difficulties, including notably: delayed pharyngeal swallow (73.2%) and faulty oro-pharyngeal transport (53.6%) which can translate into vallecular residues. It also evidences a very high frequency of alteration in the mechanisms protecting the airways (89% of which 70% has no protection at all at the laryngeal level, with no difference between the two phenotypes). Moreover, the study by Taniguchi [27], evidences a large number of esophageal anomalies (such as stagnation of foods in the esophagus, reduction in esophageal peristalsis, hypomobility of the distal esophagus, etc.). In our study, 34% of patients presented an esophageal transit anomaly on the videofluoroscopy of swallowing, but few patients underwent this examination and none had a manometry, which constitutes a limitation.

Finally, in terms of the consequences of the swallowing difficulties, the study by Do et al. [28] assessed the median interval between the start of the dietary modifications and the onset of MSA symptoms, and then with the onset of dysphagia: the two intervals were found to be longer in MSA-C. On the other hand, there was no significant difference between the two phenotypes in terms of the interval between the introduction of enteral feeding after the onset of MSA symptoms and the dysphagia. In our study, it was difficult to measure these parameters since we did not have the time intervals and dates of onset for each of the symptoms. However, we showed that the phenotype does not influence the severity, the type of diet nor the introduction of nutritional supports.

Our study does not allow an accurate analysis of the links between speech production disorders (dysphonia and/or dysarthria), swallowing disorders and laryngeal abnormalities. We found a significant correlation between speech production and swallowing difficulties gathered by the self-questionnaires and the absence of a significant difference between the severity of swallowing disorder or pneumopathies and the presence of laryngeal abnormality.

Thus, if there is a link between patients' feelings for swallowing disorders and speech production disorders, these results do not allow us to hypothesize a link through laryngeal abnormalities. We did not find available information in the literature regarding this point.

***For the prognostic factors,*** several studies found an association between dysphagia and survival in patients with MSA, without this always being due to wrong-way swallows [7, 24, 29]. The first retrospective study [7] (*n* = 83) concluded that when the patients experienced early autonomic dysfunction associated with severe dysphagia, their survival time was shorter. The second was a prospective French study on 86 patients. In this study, the swallowing difficulties were only diagnosed by questioning the patients, but it showed that survival time was shorter if dysphagia was present (RR = 2.56) without giving details on the degree of severity [24]. But another retrospective study [30] (also cited in the 2021 literature review [11]) only including patients with MSA-C (*n* = 66), stated that dysphagia (prevalence of 78%) was not an independent predictive factor for mortality, but that no other clinical sign was either.

In our study, we did not evidence of any swallowing parameters that were statistically linked to survival. However, changes to the diet in terms of solid food and loss of protection mechanism for the upper airways did tend to have an impact on a more rapid aggravation of UMSARS I + II.

On this question of prognosis, our results corroborate those of the Bologna conference [10] which concluded that (1) dysphagia is proven to be associated with poor survival, (2) aspiration is a consequence of dysphagia and impacts survival, (3) there is no proof that the specific characteristics of dysphagia affect survival.

Our results allow us to make the following hypothesis: the loss of upper airway protection mechanisms associated with vocal cord movement anomalies plays a role in the occurrence of aspiration pneumonia, the leading cause of death. Indeed, this loss of protection induces aspiration of endogenous secretions (saliva, gastric reflux), which can be responsible for pneumopathies. This observation could explain why severe swallowing difficulties were not identified as isolated prognostic factors in our study. This provides additional backup for the hypothesis made by Benarroch in 1993 on the link between dysautonomia and ventilation problems by a poor ventilation response and poor cardio-respiratory adaptation on hypoxia and hypercapnia [31], factors that increase the desynchronization of respiration/swallowing which is needed to protect the airways alongside the triggering of reflexes to swallow endogenous secretions.

All of these results thus reveal that particularity of MSA is a Pharyngolaryngeal defect prevalent in the ventilation function comprising a loss of expulsion mechanisms such as coughing and of “reflex” swallowing of endogenous secretions or food residues, and not only food swallowing.

In all cases, early care provision for the ventilation consequences (adapted non-invasive ventilation) and nutritional complications could contribute to improving the prognosis. This observation leads us to propose that a pharyngolaryngeal ENT examination is systematically offered in MSA care provision. This type of care appears to be even more appropriate than the proposition made by Higo et al. [26] to practice systematic videofluoroscopy and manometry monitoring for patients suffering from MSA for over 5 years. Finally, the notion of a nighttime or daytime inspiration noise should lead to a referral for Pharyngolaryngeal assessment.

## Conclusion

This study allowed us on one hand to underline the Pharyngolaryngeal semiological profile of MSA, which is useful for ENT clinicians: laryngeal mobility problems mainly in abduction, notably at rest (on the respiratory function), a defect in the upper airway protection mechanisms; swallowing defects: mainly moderate problems whose main mechanisms are delayed pharyngeal swallow and/or faulty oropharyngeal transport.

On the other hand, we were able to identify prognostic factors that could allow us to predict the progression of the MSA, which could impact the multi-disciplinary decision-making. These mainly involve laryngeal movement anomalies. Considering these results, it would be interesting for ENT specialists to test the protection mechanisms of the upper airways, whose absence is very frequent in MSA and which can have a negative effect on the prognosis. We thus conclude that MSA is a neurodegenerative pathology with a poor prognosis which induces Pharyngolaryngeal signs that can sometimes be identified early on. The ENT specialist should carry out a detailed Pharyngolaryngeal examination to search for neurological signs that could raise the suspicion of MSA. Indeed, early detection makes it possible to provide rapid and appropriate care for respiratory and nutritional complications.

## References

[1] Roberts M (2019) Etude polysomnographique des troubles respiratoires et de la dysautonomie au cours du sommeil dans l’atrophie multisystématisée. http://thesesante.ups-tlse.fr/2891/. (**Published online October 1, 2019. Accessed Apr 6, 2021**)

[2] Fanciulli A, Wenning GK (2015). Multiple-system atrophy. N Engl J Med.

[3] Low PA, Reich SG, Jankovic J (2015). Natural history of multiple system atrophy in the USA: a prospective cohort study. Lancet Neurol.

[4] Coon EA, Sletten DM, Suarez MD (2015). Clinical features and autonomic testing predict survival in multiple system atrophy. Brain.

[5] Starhof C, Korbo L, Lassen CF, Winge K, Friis S (2016). Clinical features in a Danish population-based cohort of probable multiple system atrophy patients. Neuroepidemiology.

[6] Meissner WG, Fernagut P-O, Dehay B (2019). Multiple system atrophy: recent developments and future perspectives. Mov Disord.

[7] O’sullivan SS (2021). Clinical outcomes of progressive supranuclear palsy and multiple system atrophy. Brain.

[8] Foubert-Samier A, Pavy-Le Traon A, Guillet F (2020). Disease progression and prognostic factors in multiple system atrophy: a prospective cohort study. Neurobiol Dis.

[9] Gilman S (2008). Second consensus statement on the diagnosis of multiple system atrophy. Neurology.

[10] Cortelli P, Calandra-Buonaura G, Benarroch EE (2019). Stridor in multiple system atrophy: consensus statement on diagnosis, prognosis, and treatment. Neurology.

[11] Calandra-Buonaura G, Alfonsi E, Vignatelli L (2021). Dysphagia in multiple system atrophy consensus statement on diagnosis, prognosis and treatment. Parkinsonism Relat Disord.

[12] Wenning GK, Tison F, Seppi K (2004). Development and validation of the Unified Multiple System Atrophy Rating Scale (UMSARS). Mov Disord.

[13] Johnson A, Jacobson B, Grywalski C, Silbergleit A, Jacobson G, Benninger M (1997). (1997) The Voice Handicap Index (VHI): development and validation. Am J Speech Lang Pathol.

[14] Balaguer M, Farinas J, Fichaux-Bourin P, Puech M, Pinquier J, Woisard V (2020). Validation of the French versions of the speech handicap index and the phonation handicap index in patients treated for cancer of the oral cavity or oropharynx. Folia Phoniatr Logop.

[15] Fichaux-Bourin P, Woisard V, Grand S, Puech M, Bodin S (2009). Validation d’un questionnaire d’auto-evaluation de la parole (Parole Handicap Index). Rev Laryngol Otol Rhinol (Bord).

[16] Woisard V, Andrieux MP, Puech M (2006). Validation of a self-assessment questionnaire for swallowing disorders (Deglutition Handicap Index). Rev Laryngol Otol Rhinol (Bord).

[17] Speyer R, Cordier R, Bouix C, Gallois Y, Woisard V (2021). Using classical test theory to determine the psychometric properties of the Deglutition Handicap Index. Dysphagia.

[18] Gao C, Liu J, Tan Y, Chen S (2020). Freezing of gait in Parkinson’s disease: pathophysiology, risk factors and treatments. Transl Neurodegener.

[19] Wenning GK, Geser F, Krismer F (2013). The natural history of multiple system atrophy: a prospective European cohort study. Lancet Neurol.

[20] Grimaldi S, Renaud M, Robert D (2020). Prevalence and characterisation of vocal fold motion impairment (VFMI) in patients with Multiple system atrophy compared with Parkinson’s disease. Rev Neurol (Paris).

[21] Tipton PW, Ekbom DC, Rutt AL, van Gerpen JA (2020). Vocal fold “Paralysis”: an early sign in multiple system atrophy. J Voice.

[22] Warnecke T, Vogel A, Ahring S (2019). The shaking palsy of the larynx-potential biomarker for multiple system atrophy: a pilot study and literature review. Front Neurol.

[23] Gandor F, Vogel A, Claus I (2020). Laryngeal movement disorders in multiple system atrophy: a diagnostic biomarker?. Mov Disord.

[24] Krim E, Yekhlef F, Chrysostome V, Ghorayeb I, Tison F (2007). Atrophie multisystématisée : survie et facteurs pronostiques dans la cohorte « MSA-Aquitaine ». Revue Neurologique.

[25] Palma J-A, Vernetti PM, Perez MA (2021). Limitations of the Unified Multiple System Atrophy Rating Scale as outcome measure for clinical trials and a roadmap for improvement. Clin Auton Res.

[26] Lee HH, Seo HG, Kim K-D (2018). Characteristics of early oropharyngeal dysphagia in patients with multiple system atrophy. Neurodegener Dis.

[27] Taniguchi H (2015). Esophageal involvement in multiple system atrophy. Dysphagia.

[28] Do HJ, Seo HG, Lee HH (2020). Progression of oropharyngeal dysphagia in patients with multiple system atrophy. Dysphagia.

[29] Müller J, Wenning GK, Verny M (2001). Progression of dysarthria and dysphagia in postmortem-confirmed parkinsonian disorders. Arch Neurol.

[30] Lieto M (2019). Longitudinal study of a cohort of MSA-C patients in South Italy: survival and clinical features. Neurol Sci.

[31] Benarroch EE, Chang FL (1993). Central autonomic disorders. J Clin Neurophysiol.

